# Multi-Compartment Profiling of Bacterial and Host Metabolites Identifies Intestinal Dysbiosis and Its Functional Consequences in the Critically Ill Child

**DOI:** 10.1097/CCM.0000000000003841

**Published:** 2019-08-15

**Authors:** Anisha Wijeyesekera, Josef Wagner, Marcus De Goffau, Sarah Thurston, Adilson Rodrigues Sabino, Sara Zaher, Deborah White, Jenna Ridout, Mark J. Peters, Padmanabhan Ramnarayan, Ricardo G. Branco, M. Estee Torok, Frederic Valla, Rosan Meyer, Nigel Klein, Gary Frost, Julian Parkhill, Elaine Holmes, Nazima Pathan

**Affiliations:** 1 Department of Human Microbiome Studies, University of Reading, Berkshire, United Kingdom.; 2 Wellcome Sanger Institute, Cambridge, Cambridgeshire, United Kingdom.; 3 Department of Paediatrics, University of Cambridge, Cambridge, United Kingdom.; 4 Federal University of Alagoas, Maceio, Brazil.; 5 Paediatric Intensive Care Unit, Cambridge University Hospitals NHS Foundation Trust, Cambridge, United Kingdom.; 6 Paediatric Intensive Care Unit, Great Ormond Street Hospital, London, United Kingdom.; 7 UCL Institute of Child Health, Respiratory, Critical Care and Anaesthesia Section, London, United Kingdom.; 8 St Mary’s Hospital, London, United Kingdom.; 9 Hospices Civils de Lyon, Pediatric Intensive Care, Hôpital Femme Mère Enfant, Lyon-Bron, France.; 10 Imperial College London, London, United Kingdom.

**Keywords:** 16S ribosomal ribonucleic acid gene sequencing, critical illness, critically ill child, gut health, intestinal microbiome, metabolomics

## Abstract

**Objectives:**

Adverse physiology and antibiotic exposure devastate the intestinal microbiome in critical illness. Time and cost implications limit the immediate clinical potential of microbial sequencing to identify or treat intestinal dysbiosis. Here, we examined whether metabolic profiling is a feasible method of monitoring intestinal dysbiosis in critically ill children.

**Design:**

Prospective multicenter cohort study.

**Setting:**

Three U.K.-based PICUs.

**Patients:**

Mechanically ventilated critically ill (*n* = 60) and age-matched healthy children (*n* = 55).

**Interventions:**

Collection of urine and fecal samples in children admitted to the PICU. A single fecal and urine sample was collected in healthy controls.

**Measurements and Main Results:**

Untargeted and targeted metabolic profiling using 1H-nuclear magnetic resonance spectroscopy and liquid chromatography-mass spectrometry or urine and fecal samples. This was integrated with analysis of fecal bacterial 16S ribosomal RNA profiles and clinical disease severity indicators. We observed separation of global urinary and fecal metabolic profiles in critically ill compared with healthy children. Urinary excretion of mammalian-microbial co-metabolites hippurate, 4-cresol sulphate, and formate were reduced in critical illness compared with healthy children. Reduced fecal excretion of short-chain fatty acids (including butyrate, propionate, and acetate) were observed in the patient cohort, demonstrating that these metabolites also distinguished between critical illness and health. Dysregulation of intestinal bile metabolism was evidenced by increased primary and reduced secondary fecal bile acid excretion. Fecal butyrate correlated with days free of intensive care at 30 days (*r* = 0.38; *p* = 0.03), while urinary formate correlated inversely with vasopressor requirement (*r* = –0.2; *p* = 0.037).

**Conclusions:**

Disruption to the functional activity of the intestinal microbiome may result in worsening organ failure in the critically ill child. Profiling of bacterial metabolites in fecal and urine samples may support identification and treatment of intestinal dysbiosis in critical illness.

## Abstract 2

Supplemental Digital Content is available in the text.

There has been a surge of interest in the contribution of the gut microbiome to health and disease. Bacteria residing in the intestinal tract are, in health, compartmented from the host but have a symbiotic relationship, contributing to metabolic, endocrine, and immune functions.

The composition and diversity of the intestinal microbiome in critical illness is likely to be impacted by poor intestinal perfusion, hypoxia, lack of enteral feeds, and antimicrobial therapy (1–4). This creates opportunities for the proliferation of potentially pathogenic species associated with adverse outcomes, including secondary infection and mortality (5–9).

Profiling of the intestinal microbiome has generally been undertaken through sequencing of microbial DNA. However, more detailed functional information about the intestinal microbiome is possible through capture of metabolic outputs. ^1^H-nuclear magnetic resonance (^1^H-NMR) spectroscopy and mass spectrometry (MS) allow simultaneous detection of both human and microbial metabolites within biological samples. This may offer an insight into host-microbe interactions in the complex human system. Furthermore, such methods could provide a more cost-effective approach to understanding the clinical impact of intestinal dysbiosis in critical illness.

We previously identified significant plasma metabolic signatures of critical illness and organ failure in critically ill children (10). In the current study, we set out to characterize the functional capacity of the intestinal microbiome in critical illness, through multi-compartmental metabolic profiling. We examined changes in bacterial and host metabolites in urine and feces in order to characterize the gut microbiota-host relationship in severe illness.

## MATERIALS AND METHODS

### Study Population

Critically ill children between 1 and 16 years old were consecutively enrolled at the time of admission to the PICU if they were mechanically ventilated and an admission urine sample was available within 48 hours of PICU admission. Children who were on chronic steroid or immune suppressant treatment were excluded. Data were obtained from the clinical health records. Healthy children, recruited from the local community, were eligible if they were well, had a normal healthy diet, and had not received antibiotics in the prior 3 months.

Approval for the study was granted by the East Midlands-Nottingham 2 Research Ethics Committee for recruitment from the PICUs at Cambridge University Hospitals NHS Foundation Trust, Great Ormond Street Hospital NHS Foundation Trust, and Imperial College Healthcare NHS Foundation Trust and from the City Road and Hampstead Research Ethics committee for recruitment of healthy children in the Cambridge vicinity. Parental informed consent was obtained prior to participation in the study.

### Samples

Urine samples were collected via indwelling catheters as soon as possible after admission to PICU (timepoint 1). Further samples were obtained at day 3–5 and days 6–8 (timepoint 2 and timepoint 3, respectively) after admission. Urine samples from healthy children were collected directly into 20 mL universal containers and stored at –70°C until use.

Fecal samples were collected from early (within first 2 d of PICU admission) and late (days 5–8 of PICU admission) in critically ill children, and a single sample obtained from healthy children. Samples were collected from nappies, placed in sterile plastic containers and stored at –70°C until use.

### Clinical Data Acquisition

Disease severity was defined through collation of bedside physiologic data. In addition, routinely collected clinical data were recorded for multivariable analysis to identify associations with cardiovascular failure (the maximal inotrope score [11]), respiratory failure (days free of mechanical ventilation at 30 d), and critical illness duration (days free of PICU at 30 d).

### ^1^H-NMR Spectroscopy

Fecal water and urine samples were prepared for ^1^H-NMR Spectroscopy according to published protocols (12). Acquired spectroscopic data were processed using the TopSpin 3.1 software package (Bruker Biospin, Rheinstetten, Germany). Data processing was undertaken using Matlab (Version 8.3.0.532 R2014a; Mathworks, Natick, MA). Further details are given in **supplemental methods** (Supplemental Digital Content 1, http://links.lww.com/CCM/E693).

### Chemometric Analysis of Spectroscopic Data

Processed spectroscopic data were imported to the SIMCA 13.0 software package (Umetrics AB, Umeå, Sweden) to conduct unsupervised multivariate statistical analysis. Principal components analysis was used to evaluate similarities/differences in urinary and fecal metabolite composition between groups. The *R*^2^ and *Q*^2^ variables provided an indication of goodness of fit (*R*^2^) as well as goodness of prediction (*Q*^2^) of the models. Supervised Orthogonal Projections to Latent Structures Discriminant Analysis models were calculated using one predictive and two orthogonal components. The models were assessed based on variance explained (*R*^2^Y) and predictive ability (*Q*^2^Y) metrics. Further details are given in the supplemental methods (Supplemental Digital Content 1, http://links.lww.com/CCM/E693).

### Bile Acid Quantification

Bile acid data were acquired using an liquid chromatography-mass spectrometry quadrupole time-of-flight instrument in profiling mode, according to protocols described by Sarafian et al (13). The identity of the bile acids was confirmed by comparison of their retention times and mass spectra with those of reference standards also included in the analytical run.

### Fecal DNA Extraction and Bacterial 16S Ribosomal RNA Gene Sequencing

Whole microbial genome DNA was extracted from fecal samples using PowerFecal DNA Isolation Kit (Mo Bio Laboratories, Carlsbad, CA). Aliquots of extracted genomic DNA were quantified using Qubit dsDNA HS Assay Kit (Life Technologies, Waltham, MA). The DNA was amplified with Illumina adapter and indexed polymerase chain reaction primers. Bacterial 16S ribosomal RNA was sequenced using the Illumina MiSeq sequencing platform (Illumina, Inc., San Diego, CA) as previously described (14). Further details are given in the supplemental methods (Supplemental Digital Content 1, http://links.lww.com/CCM/E693).

### Bioinformatic and Statistical Analysis for Bacterial 16S Sequence Data

Multivariate diversity analysis between patient and control samples was performed using PERmutational Multivariate ANalysis Of VAriance (PERMANOVA) using the Adonis function from the R package VEGAN (15).

Rank-based indirect gradient analysis “nonmetric multidimensional scaling (NMDS)” was used for the visualization of taxonomic differences between the different groups, using metMDS in R (15, 16). NMDS attempts to represent, as closely as possible, the pairwise dissimilarity between objects in a low-dimensional space. Further details are given in the supplemental methods (Supplemental Digital Content 1, http://links.lww.com/CCM/E693).

### Statistical Analysis

Multiple linear and logistic regression analyses were applied using the Statistical Package for the Social Sciences Version 21 (SPSS Statistics 22, IBM Corp., Armonk, NY). Categorical variables were analyzed using chi-square and Fisher exact tests as appropriate. Continuous variables were compared using two-sided *t* tests (for parametric variables) and the Mann-Whitney *U* test (nonparametric variables). A false discovery rate (FDR) was used to adjust for multiple metabolite testing. Metabolites with FDR less than 0.05 were considered significant.

Multivariate beta diversity analysis between groups, including age, gender, and diagnostic category, was performed using R studio and the Statistical Package for the Social Sciences (SPSS Statistics 22, IBM Corp.). All presented *p* values were corrected for multiple comparisons using the Benjamini-Hochberg FDR method.

## RESULTS

### Participant Demographics

The mean (sd) ages and weights were 70.28 months (52.8 mo) and 21.5 kg (13.5 kg) for patients; 73.5 months (43.7 mo) and 20.9 kg (10.1–41 kg) for controls. Four children died in the patient cohort (5.9%). All the critically ill children had received one or more broad-spectrum antibiotics at the time of sampling. Further clinical details and antibiotic exposure are given in **Table 1**.

### Urinary Metabolic Profiling Demonstrates Loss of Intestinal Bacterial Metabolic Activity in Critical Illness

**Figure 1.**
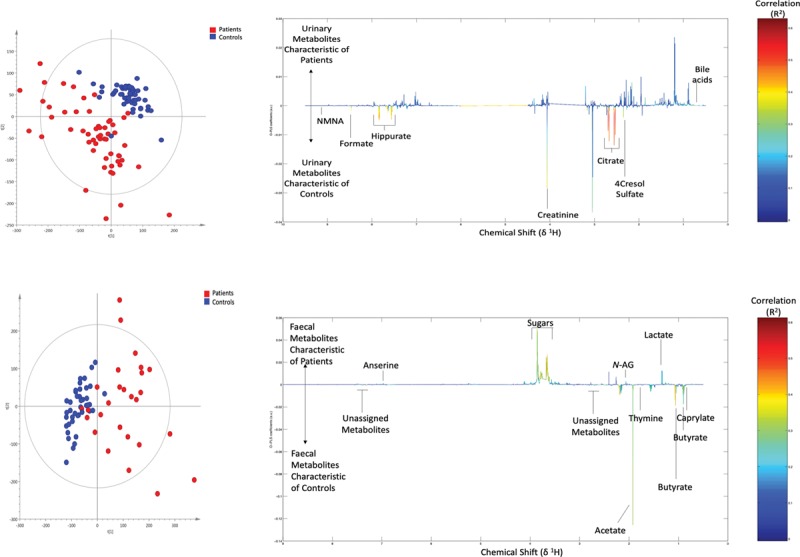
Urinary and fecal ^1^H-nuclear magnetic resonance (^1^H-NMR) global metabolic profiles of critically ill and healthy children. **A**, Unsupervised principal components analysis (PCA) scores plot of admission urine samples from age-matched critically ill (*red*) and healthy (*blue*) children. *R*^2^ = 0.16, *Q*^2^ = 0.09. **B**, Supervised Orthogonal Projections to Latent Structures Discriminant Analysis (O-PLS-DA) loadings line plot. Urinary metabolites higher in critically ill children (*up*) compared with age-matched healthy children (*down*). The *color bar* indicates the correlation coefficient (*R*^2^) (i.e., the *redder* the peak, the higher the correlation). *R*^2^Y = 0.89, *Q*^2^Y = 0.80. **C**, Unsupervised PCA scores plot of fecal samples from age-matched critically ill (*red*, *n* = 27) and healthy control (*blue*, *n* = 41) samples. *R*^2^ = 0.23, *Q*^2^ = 0.08. **D**, Supervised O-PLS-DA loadings line plot. Fecal metabolites higher in critically ill children (*up*) compared with age-matched healthy children (*down*). *R*^2^Y = 0.96, *Q*^2^Y = 0.85. *R*^2^Y = variance explained, *Q*^2^Y= predictive ability. a.u = arbitrary units, N-AG = n-acetylglucosamine, NMNA = n-nethylnicotinamide.

We identified robust differences in the global ^1^H-NMR metabolic profiles of admission (timepoint 1) urine samples from critically ill compared with healthy children (****Fig. 1*A*****), indicating differences in both endogenous and intestinal microbiome-derived metabolites. Using supervised multivariate statistical analysis, we observed *R*^2^Y and *Q*^2^Y values of the generated model at 0.89 and 0.8, respectively (**Fig. 1*B***).

Urinary excretion of several mammalian-microbial co-metabolites were reduced in critically ill patient samples (**Supplementary Table 1**, Supplemental Digital Content 2, http://links.lww.com/CCM/E694). These included hippurate, 4-cresol sulphate, and formate. In addition, two bile acid peaks were associated with samples from the patient cohort, and a strong reduction in urinary citrate excretion observed in samples from critically ill compared with healthy children.

We did not observe a strong separation in the global urine metabolic profile based on sample timing during the course of PICU stay (**Supplementary Fig. 1**, ***a*** and ***b***, Supplemental Digital Content 3, http://links.lww.com/CCM/E695; **legend**, Supplemental Digital Content 9, http://links.lww.com/CCM/E701). There was no difference in global metabolic profiles based on gender, comorbidity status, duration or class of antibiotic exposure, and survival.

### Untargeted Fecal Metabolic Profiling Indicates Loss of Bacterial Fermentation Activity in Critical Illness

As with urinary metabolic profiles, the global ^1^H-NMR metabolic profiles of fecal water in critically ill compared with healthy children were strongly separated (**Fig. 1*C***). Using supervised multivariate statistical analysis, we observed *R*^2^Y and *Q*^2^Y values of the generated model at 0.7 and 0.17, respectively (**Fig. 1*D***). Metabolites showing discrimination between critically ill and healthy samples included short-chain fatty acid (SCFA) concentrations (among them butyrate, acetate, and propionate) and the nucleobase thymine which associated with healthy child sample profiles. Lactate and *N*-acetylglycoprotein associated with samples from critically ill children (**Supplementary Table 2**, Supplemental Digital Content 4, http://links.lww.com/CCM/E696).

As with the urine, we did not identify differences based on gender, comorbidities, duration or class of antibiotic exposure, and survival status.

### Intestinal Bacterial Metabolism of Bile Acids Is Compromised During Critical Illness

**Figure 2.**
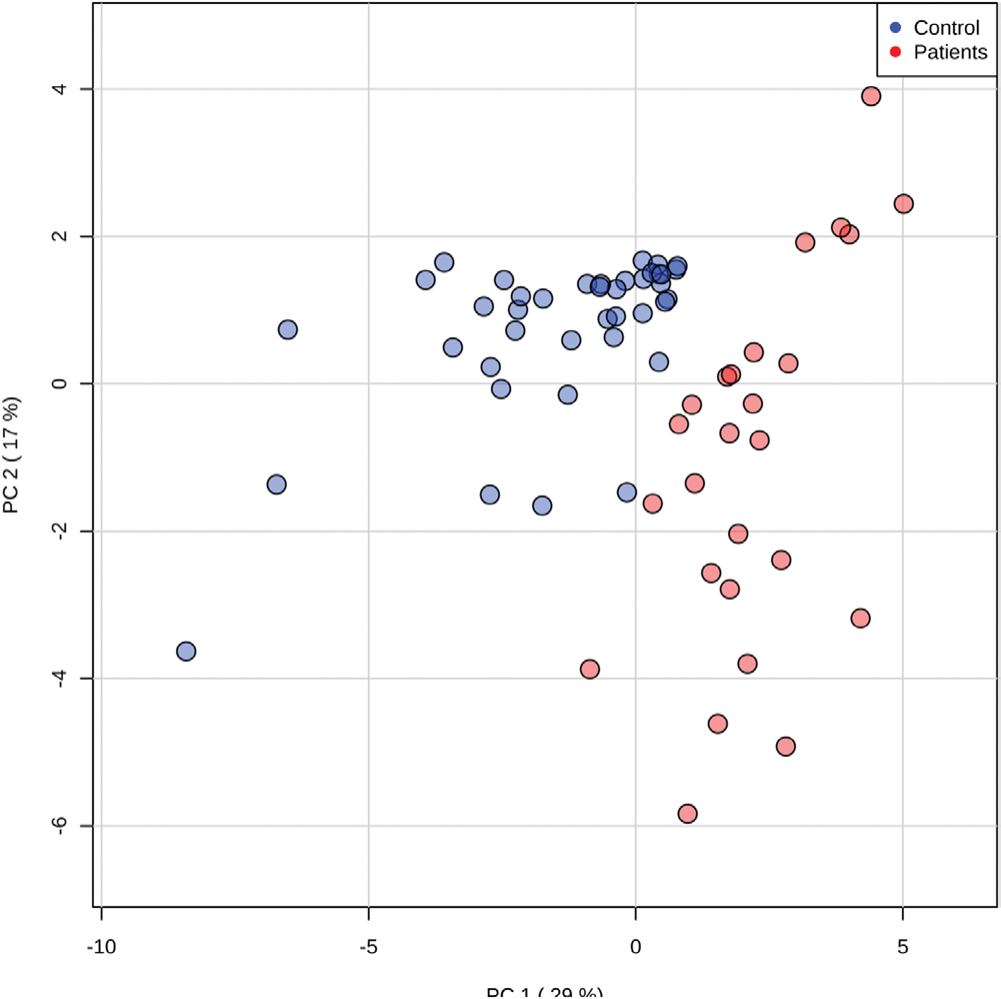
Fecal liquid chromatography-mass spectrometry bile acid (BA) profiles of critically ill and healthy children. **A**, Unsupervised principal components analysis scores plot of critically ill (*red*) and healthy control (*blue*) samples. *R*^2^ = 0.47, *Q*^2^ = 0.16. **B**, Changes in the metabolism of BAs in critically ill (in *red*) compared with healthy children (in *blue*) illustrate accumulation of primary BAs and a reduction in lithocholic acid in critically ill children. 5β-CA-3β, 12a-diol = 5β-cholanic acid-3β, 12a-diol, FDR 2.952E-6, 23 nor 5β- CA-3α, 12a-diol = 23-nor-5b-cholanic acid-3a, 12a-diol, FDR 1.664E-7, 3KCA = 3 ketocholanic acid, FDR 3.505E-7, 3a-H-12 KLCA = 3a-hydroxy-12 ketolithocholic acid, FDR 4.174E-6, CA = cholic acid, FDR 2.142E-7, DCA = deoxycholic acid, FDR 0.004, FDR = false discovery rate, ILCA = isolithocholic acid, FDR 1.596E-10, LCA = lithocholic acid, FDR 6.157E-10, PC = principal component, TCA = taurocholic acid, FDR 0.001.

Based on the presence of increased urinary bile acid concentrations in patient urine samples, we went on to examine intestinal bile acid metabolism using targeted MS. We observed clear separation in overall fecal bile acid profile between critically ill and healthy children (****Fig. 2*A*****), with observed *R*^2^Y and *Q*^2^Y values of the generated model at 0.47 and 0.27, respectively (**Supplementary Fig. 2**, Supplemental Digital Content 5, http://links.lww.com/CCM/E697; legend, Supplemental Digital Content 9, http://links.lww.com/CCM/E701).

In samples from critically ill children, a statistical increase in primary bile acid concentrations (cholic and choledeoxycholic acids) was observed. A reduction in concentration of secondary bile acids dependent on commensal bacterial metabolism was observed, including deoxycholic and lithocholic acids along with other bile acid metabolites arising from bacterial dehydroxylation, epimerization, or oxidation (**Fig. 2*B***).

### Loss of Intestinal Metabolic Capacity Is Associated With Intestinal Dysbiosis in the Critically Ill Child

Using the Shannon alpha diversity index as an indicator of fecal microbial diversity, we observed mean (sd) alpha diversity at genus level of 2.82 (0.34) compared with 2.12 (0.87) in samples from healthy versus critically ill children, respectively (*p* < 0.0001).

**Figure 3.**
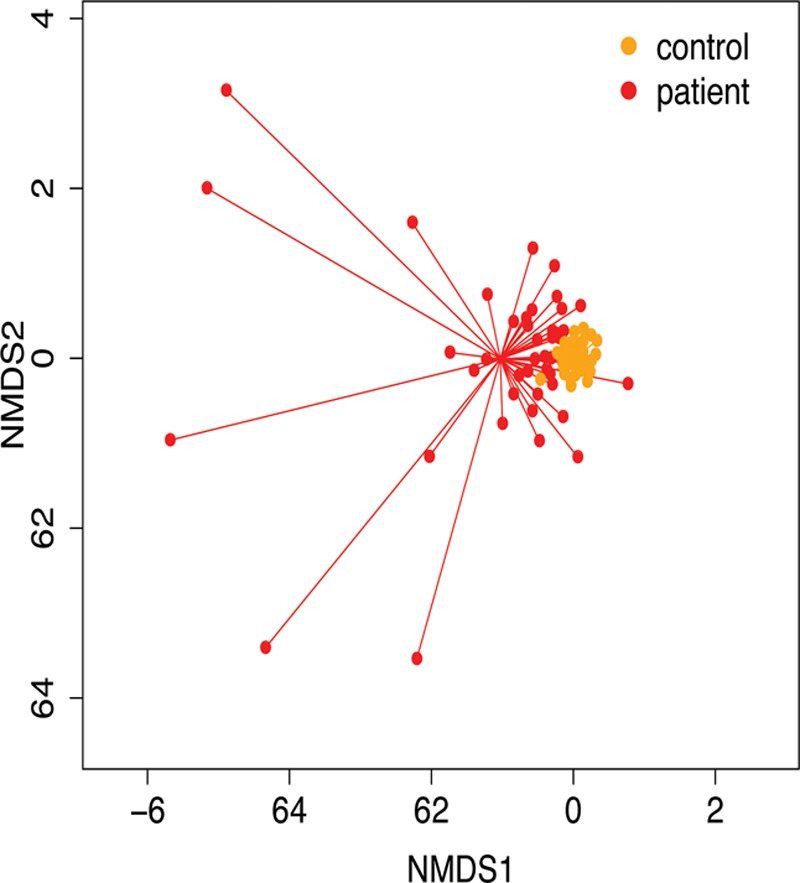
Bacterial composition in age-matched critically ill and healthy children. Nonmetric multidimensional scaling (NMDS) plot of fecal microbial composition at genus level. High variability is seen in samples from critically ill children (*red*) compared with healthy controls (*orange*). PERmutational Multivariate ANalysis Of Variance (PERMANOVA) *p* = 0.0001, *F* = 3.314. Samples from healthy children were tightly clustered together, suggesting greater similarity in composition, while those from patients were scattered widely across the plot and statistically separated from the healthy profiles (PERMANOVA test: *F* = 9.78; *p* < 0.001).

NMDS demonstrated greater inter-individual variability in patient 16S profiles compared with those of healthy children (****Fig. 3****). Hierarchical analysis demonstrated that healthy children had higher prevalence of Bacteroides, Faecalibacterium, and Ruminococcus genera. Samples from critically ill children showed increased presence of noncommensals (e.g., Enterococcus and Streptococcus), and of normally low-prevalence microbial genera (**Supplementary Fig. 3**, Supplemental Digital Content 6, http://links.lww.com/CCM/E698; legend, Supplemental Digital Content 9, http://links.lww.com/CCM/E701).

We did not observe any statistical difference in the overall fecal microbial profile based on specific antibiotic class exposure or the days of antibiotic exposure prior to sampling. However, that the proportional abundance of the Enterococcus genus in patient samples directly correlated with the number of antibiotic classes administered prior to sample collection (*r* = 0.43; *p* = 3 × 10^–6^).

### Patterns of Change in Composition and Function of the Intestinal Microbiome in Critical Illness

We undertook integrated analysis of fecal microbial 16S, and metabolic profiles using regression analysis and unsupervised clustering. Metabolites associated with samples from healthy children included acetate, propionate, and butyrate. An inverse correlation was noted of fecal SCFA levels with proportional abundance of Enterococcus, Bifidobacteria, Escherichia-Shigella, and Staphylococcus genera (Supplementary Table 1, Supplemental Digital Content 2, http://links.lww.com/CCM/E694). Linear regression demonstrated that abundance of Faecalibacterium, Fusicatenibacter, and Phascolarctobacterium were most predictive of fecal butyrate levels (*R*^2^ = 0.32; *F* = 9.44; *p* < 0.0001).

We observed that intermediate metabolites of the citric acid cycle including fecal succinate, lactic acid, oxaloacetic acid, pyruvic, and acetoacetic acid were associated with the 16S profiles of critically ill compared with healthy children (**Supplementary Fig. 4*a***, Supplemental Digital Content 7, http://links.lww.com/CCM/E699; legend, Supplemental Digital Content 9, http://links.lww.com/CCM/E701).

We examined the association between fecal microbial composition and bile acid secretion in the patient cohort. We noted an inverse correlation between alpha diversity with primary bile acids including taurocholic acid (*r* = –0.44; *p* = 0.023) and taurohyocholic acid (*r* = –0.55; *p* = 0.003), and a direct correlation with secondary bile acids lithocholic acid (*r* = –0.48; *p* = 0.011) and isolithocholic acid (*r* = –0.6; *p* = 0.0001).

Bacterial genera including Bacteroides, Ruminococcus, Eubacterium, Lachnospiraceae, and Faecalibacterium were directly correlated with levels of secondary bile acids including deoxycholic, lithocholic, and isolithocholic acid. Conversely, these bile acids were negatively correlated with abundance of Enterococcus and Staphylococcus (**Supplementary Table 3**, Supplemental Digital Content 8, http://links.lww.com/CCM/E700).

In a linear regression model, we observed that proportional abundance of Faecalibacterium, Coprococcus, Ruminococcus, Catabacter, Enterococcus, Oscillibacter, and Pseudobutyrivibrio genera were predictive of fecal lithocholic acid concentration (*R*^2^ = 0.27; *F* = 2.34; *p* = 0.032).

### Metabolic Markers of Disease Severity in Critical Illness

The peak integral values for fecal butyrate in the early admission samples of critically ill children correlated directly with days free of intensive care at 30 days (*r* = 0.38; *p* = 0.03). Peak integral values for urinary formate were inversely correlated with vasopressor requirement as measured by the inotrope score (17) (*r* = –0.2; *p* = 0.037). Urinary citrate directly correlated with inotrope score in patient samples (*r* = –0.27; *p* = 0.004).

**TABLE 1.**
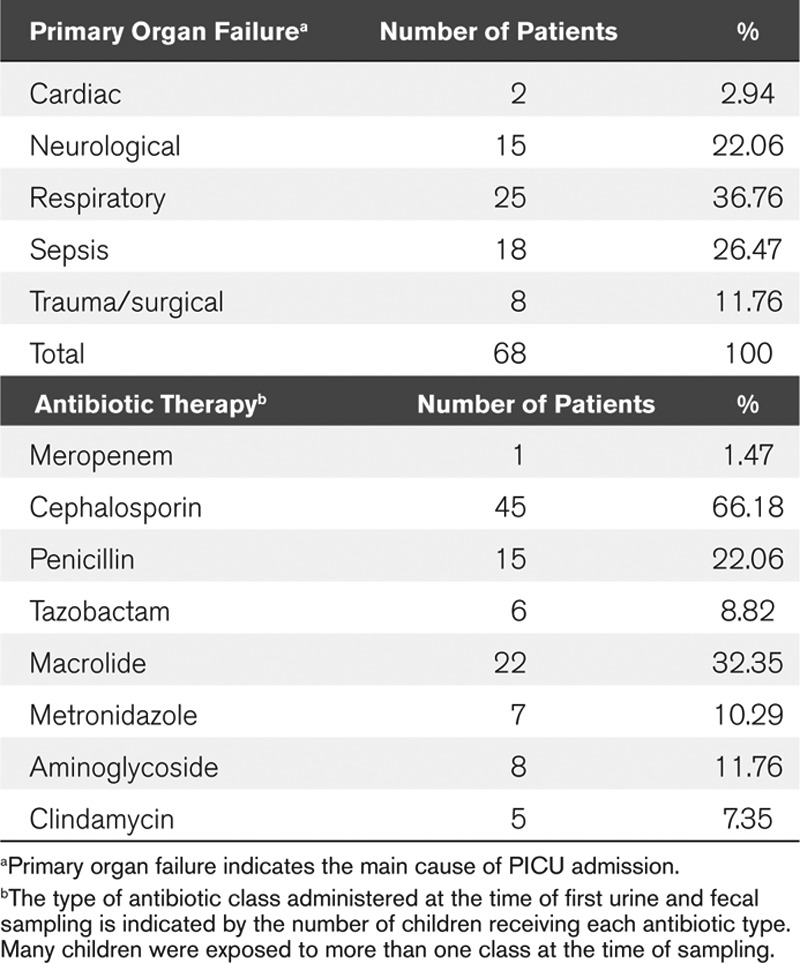
Clinical Details of Study Participants

## DISCUSSION

Although the adverse effects of critical illness on the composition of the intestinal microbiome in adults and children have been reported (5, 6, 9), the functional consequences of this on the developing pediatric microbiome have not been well described. We identified several changes in intestinal microbial activity during critical illness, including the fermentation of terminal carbohydrate metabolites into SCFAs and bile acid metabolism.

Undigested carbohydrate and protein are key substrates for fermentation by colonic microbiota, and the resulting metabolites include SCFAs, branched-chain fatty acids, ammonia, amines, phenolic compounds, and gases including hydrogen, methane, and hydrogen sulphide. A healthy trophic network of SCFAs is maintained by a diverse number of commensal species in the gut (18, 19). The three main SCFAs, acetate, propionate, and butyrate are used in a number of host metabolic processes including signaling effector roles to modulate distal organ function (20), and in maintaining the health and barrier function of the colonic mucosa (21).

Sustained reduction in fecal levels of SCFAs compared with healthy controls has been previously documented in critically ill adults (3).

Commensal microbes regulate bile acid metabolism in the lower intestine (22) and are involved in deconjugation and dehydroxylation of bile acids released by the gall bladder into the intestinal lumen (23). Ours is the first study to demonstrate dysregulation of fecal bile acid metabolism in pediatric critical illness. Our data fits with previous observations of reduced luminal SCFA and secondary bile acid concentrations in murine microbiome depletion models (24, 25).

The patient microbiome was characterized by over-representation of opportunistic pathogenic species and with species typically associated with the small intestine or with the use of antibiotics in critical illness including (*Enterococcus*) (6, 26) or with diarrhea (*Streptococcus*) (27).

We were surprised not to see any microbial or metabolic patterns of change over the course of PICU admission, given the dynamic physiology of critical illness. However, it is likely that the ongoing exposure to systemic antibiotics is likely to influence the rate of recovery of intestinal microbial activity in this population. In critically ill adults, the fecal microbiome appears to continue to show significant dysbiosis at the time of ICU discharge (29). Our patient samples were collected at or within the first few days of PICU admission, so there was little variance in duration of antibiotic exposure.

Broad-spectrum antibiotics are frequently administered to treat suspected infection in children and adults with critical illness (6, 28). Often it is done for life-saving reasons. The fact that all patients in our study received broad-spectrum antibiotics is a limitation, and future studies should include samples from nonantibiotic exposed children.

Our finding that the number of antibiotic classes administered was associated with proportional abundance of the Enterococcus genus is in keeping with studies in critically ill adults (30). The most comparable study in critically ill children (6) did not explore the issue of antibiotic class in detail, although as in our study, administration of antibiotics was widespread (89% of a population where 94% were mechanically ventilated).

We were surprised not to find a differentiation in 16S or metabolic profiles between patients with and without preexisting comorbidity. It suggests that the combination of adverse exposures in critical illness is greater than the impact of preexisting illness. Although there have been studies of the microbiome in patients with a protracted course of critical illness (26), we did not identify any studies that compared ICU admission microbiome profiles in previously well individuals compared with those living with life-long conditions.

We saw no significant impact of gender on the composition of the microbiome in our patient cohort, which was not unexpected given that the majority of children in the study were pre-pubertal. Studies in healthy neonates have not demonstrated gender-based differences in the neonatal urinary metabolome (31), and in older healthy children, population-specific factors (age, sex, body mass index, ethnicity, dietary, and country of origin) appear to be better captured in serum than in urine metabolic profiles (32). We did not find any published data of gender-based differences in critically ill children or adults. In a cohort study of 43 newborns over 2 years, the most significant influences on fecal microbiome maturation were birth mode, antibiotic exposure, and diet (33).

Our integrated analysis reflects the accumulation of intermediate fermentation metabolites in critical illness as a result of intestinal dysbiosis (**Supplementary Fig. 4*b***, Supplemental Digital Content 7, http://links.lww.com/CCM/E699; legend, Supplemental Digital Content 9, http://links.lww.com/CCM/E701). The gross decline of *Bacteroides*, *Faecalibacterium*, *Roseburia*, and *Prevotella* and their associations with the production of acetate, butyrate, and propionate substantiate this.

We did not observe any microbial or metabolic signatures of nonsurvival, although our study was not powered for this outcome. In adults, pathogen colonization of the fecal microbiome has been shown to be associated with death (9).

Fecal SCFA measurement has been shown to be an effective tool to monitor recovery of gut microbiome in neonatal and murine probiotic supplementation trials (34, 35). The ability to assess the intestinal microbiome using urine metabolic profiling is advantageous in this patient population, since fecal samples may not be available acutely. The methodology to undertake the metabolite assays is scaleable and could be used to monitor gut health during and after recovery from critical illness.

We have demonstrated that profiling of bacterial metabolites offers an insight into the functional capacity of the intestinal microbiome. A reduced abundance of these metabolites is linked to clinical disease severity.

Dietary and microbiome-based therapies are being explored for the potential to support recovery of healthy gut commensal populations during and after critical illness. The panel of bacterial metabolites we have identified could be used to stratify and monitor such interventions. Beyond critical illness, the methodology is applicable to other disorders such as inflammatory bowel disease or severe malnutrition. The technology is scalable and adapting it to a clinically relevant format is feasible.

## ACKNOWLEDGMENTS

We acknowledge the support of the Imperial College Clinical Phenotyping Centre, a core facility of the National Institute for Health Research Imperial Biomedical Research Centre’s Institute of Translational Medicine and Therapeutics. We would like to thank the children and families participating in the study, along with the clinicians treating them. We also thank the core informatics, sequencing, and pathogen informatics teams at the Wellcome Trust Sanger Institute.

## Supplementary Material
