# Supplementary material for: Multi-Compartment Profiling of Bacterial and Host Metabolites Identifies Intestinal Dysbiosis and Its Functional Consequences in the Critically Ill Child
Source: Crit Care Med. 2019 Aug 15;47(9):e727–34. doi: 10.1097/CCM.0000000000003841 (PMC6699985; doi:10.1097/CCM.0000000000003841)
Supplement: Supplementary file 1 [file ccm-47-e727-s001.docx]

**Supplemental methods**

**Chemometric analysis of spectroscopic data**

The resultant O-PLS-DA loadings line plot denotes the covariance of each data point with class (indicating direction and intensity), whilst the colorbar indicates the correlation coefficient (R^2^). Metabolites with a correlation of > 0.5 were tabulated to assess the differences in metabolic profiles between the models. Metabolites were assigned with reference to published literature assignments, online spectral compound databases (e.g. Human Metabolome DataBase (HMDB), KEGG, Biological Magnetic Resonance Data Bank) as well as in-house spectral reference libraries. Statistical correlation spectroscopic approaches (Statistical Total COrrelation SpectroscopY(1), and Subset Optimisation by Reference Matching(2)) were also employed to confirm metabolite identification.

**Fecal DNA extraction and bacterial 16S rRNA gene sequencing.**

DNA concentration were quantified using the Qubit 2.0 fluorometer and high sensitivity DNA reagents (Invitrogen). Samples were pooled in equimolar concentration and gel purified using The Wizard® SV Gel and PCR Clean-Up System (Promega). The library size was confirmed on a Tape station (Agilent Technologies) and then MiSeq sequenced using the 600 cycle MiSeq reagent kit V3, which enables 300 base pair end sequencing. The library was sequenced at the Wellcome Sanger Institute (Cambridge, United Kingdom).

All primers were purchased from Eurofins (Ebersberg, Germany). PCR reactions were pooled and purified with 75μl AMPure XP beads (Agencourt Bioscience) according to Illumina's 16S metagenomic sequencing library preparation protocol(21).

**Bioinformatic and Statistical analysis for bacterial 16S sequence data**

The heat map was generated using the R package “Heatplus” (Author Alexander Ploner, available from <https://github.com/alexploner/Heatplus)>. For the heatmap we used the Bray Curtis clustering and Ward.D method.

The hierarchical dendrogram used in the combined dendrogram/stack bar chart figure was taken from the heat map using the Heatplus package.
