# Supplementary figures and images for: Multi-Compartment Profiling of Bacterial and Host Metabolites Identifies Intestinal Dysbiosis and Its Functional Consequences in the Critically Ill Child
Source: Crit Care Med. 2019 Aug 15;47(9):e727–34. doi: 10.1097/CCM.0000000000003841 (PMC6699985; doi:10.1097/CCM.0000000000003841)

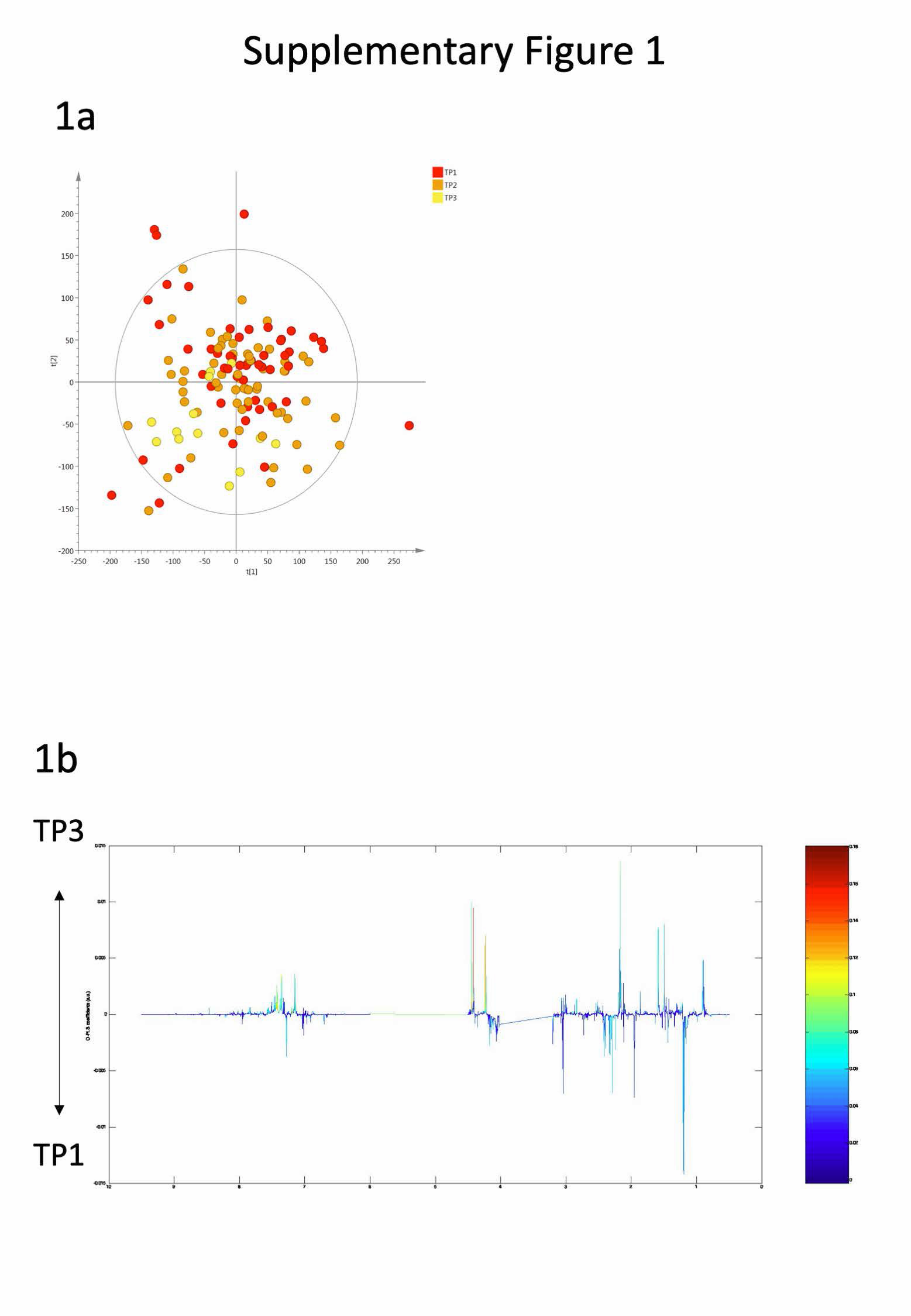

Supplement: Supplementary file 3 [file ccm-47-e727-s003.tif]

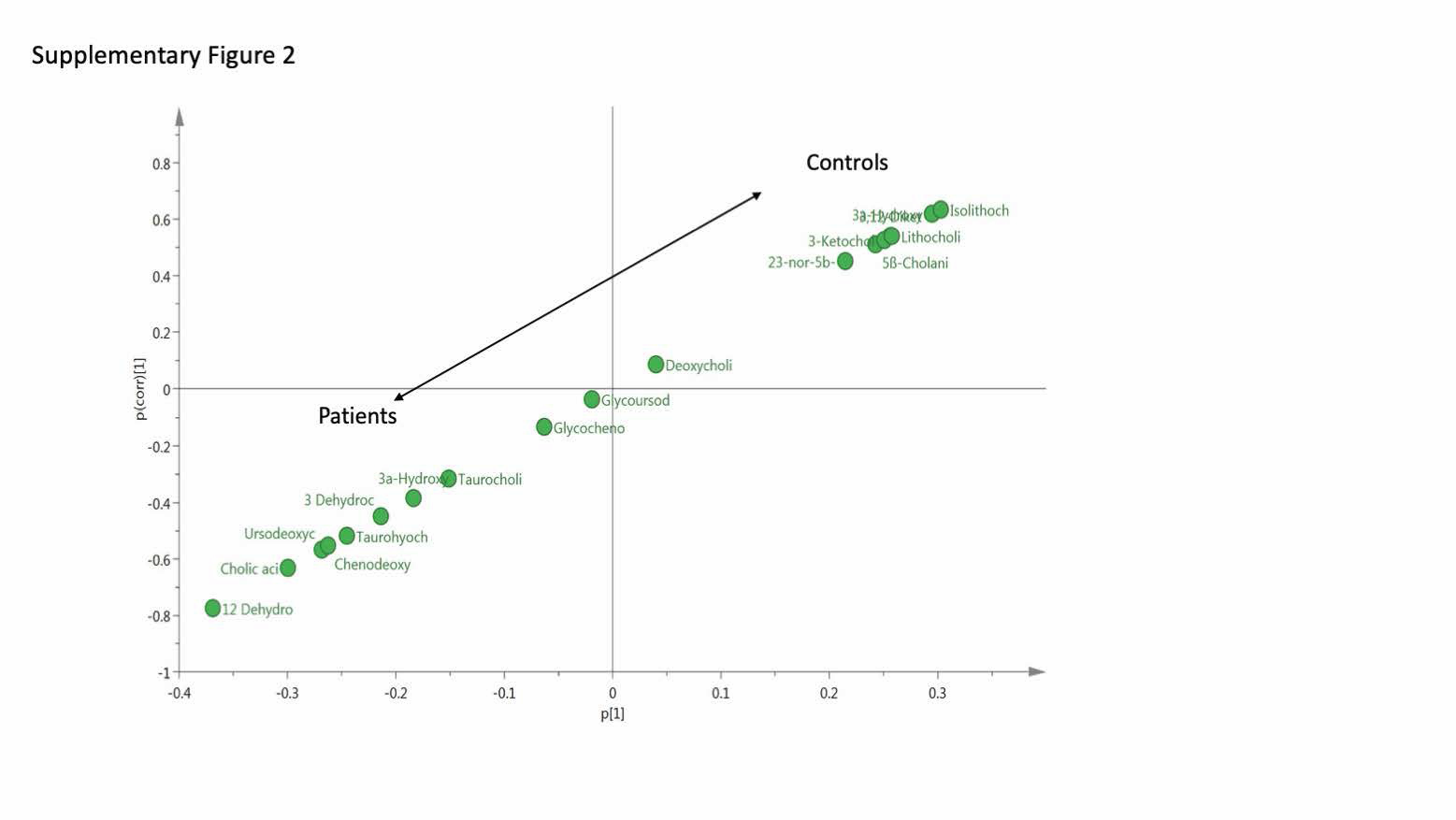

Supplement: Supplementary file 5 [file ccm-47-e727-s005.tif]

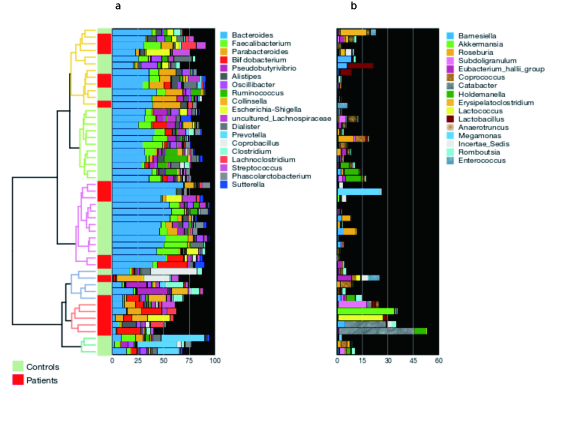

Supplement: Supplementary file 6 [file ccm-47-e727-s006.tif]

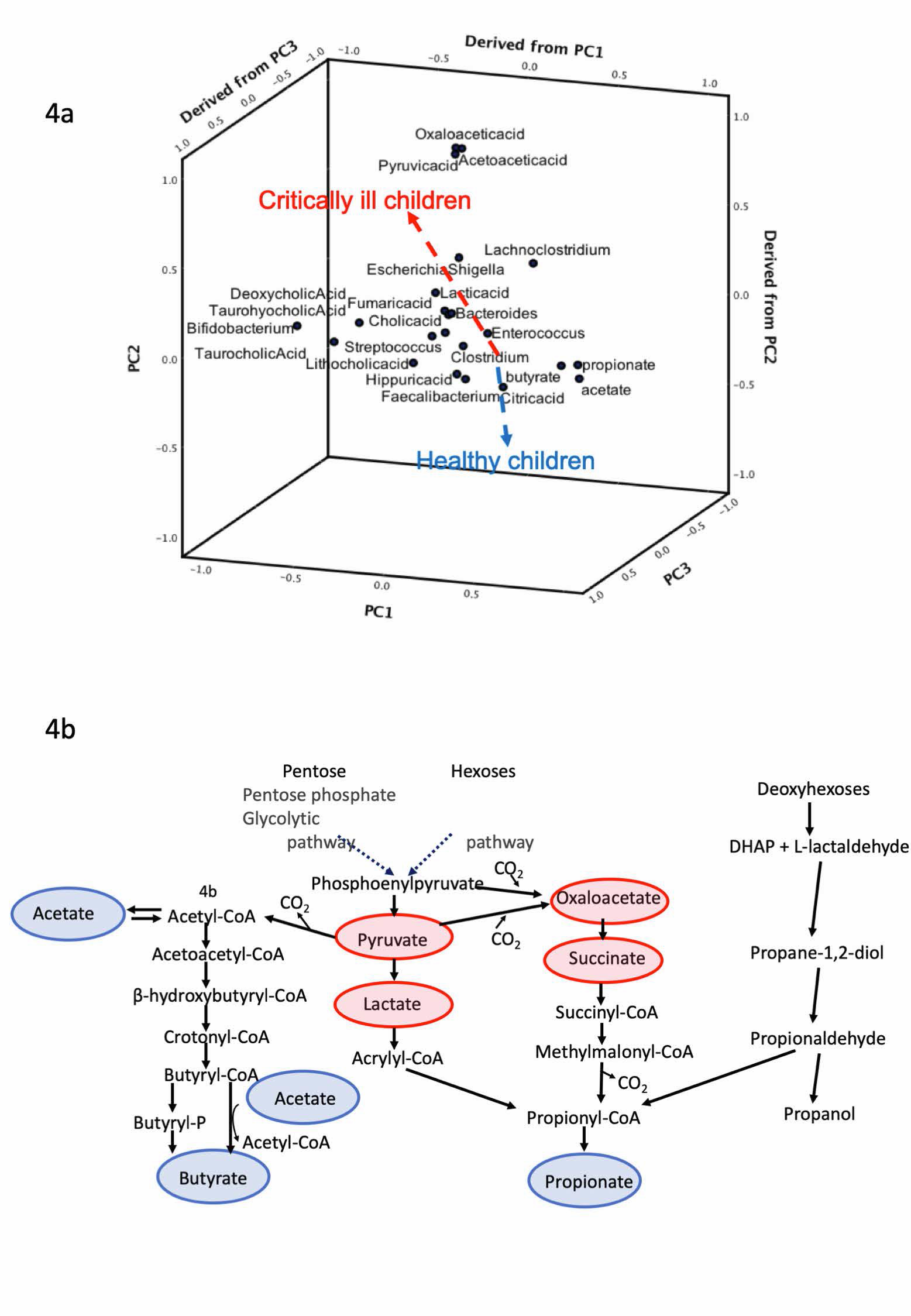

Supplement: Supplementary file 7 [file ccm-47-e727-s007.tif]
