# Supplementary material for: Multi-Compartment Profiling of Bacterial and Host Metabolites Identifies Intestinal Dysbiosis and Its Functional Consequences in the Critically Ill Child
Source: Crit Care Med. 2019 Aug 15;47(9):e727–34. doi: 10.1097/CCM.0000000000003841 (PMC6699985; doi:10.1097/CCM.0000000000003841)
Supplement: Supplementary file 4 [file ccm-47-e727-s004.docx]

Supplementary Table 2: Discriminatory fecal metabolites between patients and controls.


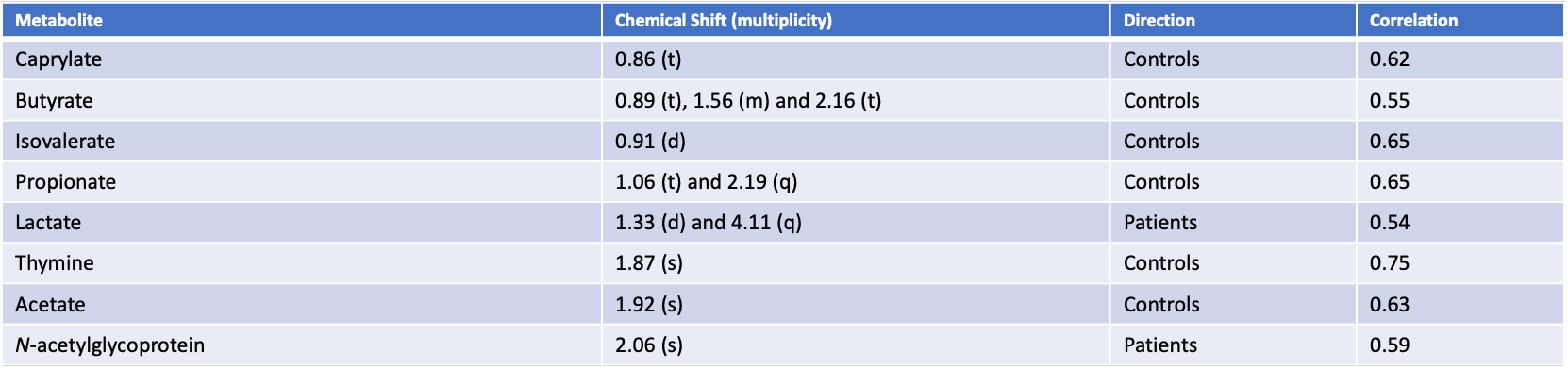


Metabolites compared by examining first fecal samples in critically ill children with samples from healthy age-matched controls.
