# Supplementary material for: Multi-Compartment Profiling of Bacterial and Host Metabolites Identifies Intestinal Dysbiosis and Its Functional Consequences in the Critically Ill Child
Source: Crit Care Med. 2019 Aug 15;47(9):e727–34. doi: 10.1097/CCM.0000000000003841 (PMC6699985; doi:10.1097/CCM.0000000000003841)
Supplement: Supplementary file 8 [file ccm-47-e727-s008.docx]

Supplementary Table 3: Bacterial genera associated with fecal bile acid abundance in critically ill children.

*indicates significance at p <0.05 ** indicates significance at p<0.001
