# Supplementary material for: Multi-Compartment Profiling of Bacterial and Host Metabolites Identifies Intestinal Dysbiosis and Its Functional Consequences in the Critically Ill Child
Source: Crit Care Med. 2019 Aug 15;47(9):e727–34. doi: 10.1097/CCM.0000000000003841 (PMC6699985; doi:10.1097/CCM.0000000000003841)
Supplement: Supplementary file 9 [file ccm-47-e727-s009.docx]

**Supplementary Figure Legends**

*Supplementary Figure 1: Timecourse analysis of urine 1H-NMR profiles from critically ill and healthy children*

Urine samples were available in the first 48 hours and then at 3-5 days after admission for 60 critically ill (42% male) and 55 healthy (50% male) children. A late urine sample was available in 13 children who remained inpatients at day 6-8.

*1a.* Unsupervised PCA scores plot of urine samples taken at Timepoint (TP)1, TP2 and TP3. R^2^=0.12, Q^2^=0.06.

*1b.* Supervised O-PLS-DA loadings line plot. Urinary metabolites higher in admission samples (TP1) (down) compared to later (TP3) samples (up). R^2^Y=0.70, Q^2^Y=0.17

*Supplementary Figure 2: Analysis of fecal bile acid concentrations in healthy and critically ill children.* Supervised O-PLS-DA loadings plot showing bile acids that are higher in healthy children (up) compared to critically ill children (down). R^2^Y=0.47, Q^2^Y=0.27.

*Supplementary Figure 3: Proportional abundance of bacterial genera in age-matched critically ill and healthy children.*

Samples were analyzed by hierarchical clustering using Bray Curtis matrix and Ward D method. Each bar in the associated stacked bar chart represents the proportional abundance of bacterial taxa at genus level. Fecal samples were obtained in 42 critically ill children (mean of 4.2 (2.5) days after admission), and in 41 healthy children from the main cohort.

**a.** Top 19 genera with a minimum abundance of 1%, covering 81% of all reads.

**b.** The next 16 top genera with a minimum abundance of 0.5%-1%, covering 10.8% of all reads.

*Supplementary Figure 4: Integrated analysis of microbial and metabolic data indicates collapse of the trophic fermentation network in critical illness.*

*4a Correlation of microbial genera with metabolic profiles.* PCA scores plot of normalized metabolite concentrations and proportional abundance of bacterial genera. Variables labelled were found to be statistically different between age-matched patients and controls. Clustering of variables associated with health and disease is observed, with separation indicated by arrows showing association with patient (red) and healthy control (blue) samples.

*4b Dysfunctional carbohydrate fermentation.*

In health, commensal species ferment carbohydrates into short chain fatty acids (highlighted in blue), such as acetate, butyrate and propionate. These were more abundant in healthy children. Depletion of these commensal bacteria in critical illness was associated with accumulation of intermediate metabolites such as succinate, pyruvate, formate and keto-acids (highlighted in red).
